# Experimental Biology, Life Science and One Health: From Basic to Translational Research

**DOI:** 10.3390/biology15211993

**Published:** 2026-11-04

**Authors:** Francesco Cappello

**Affiliations:** Department of Biomedicine, Neurosciences and Advanced Diagnostics (BiND), University of Palermo, 90127 Palermo, Italy; francesco.cappello@unipa.it

This Special Issue, “Experimental Biology, Life Science and One Health: From Basic to Translational Research”, originated in the centenary of the Italian Society of Experimental Biology, founded in 1925 at Camillo Golgi’s Institute in Pavia. This anniversary offered an opportunity to reflect on the continuing contribution of experimental biology to medicine. Understanding disease requires the study of normal biological organisation, the mechanisms through which this organisation changes, and the conditions under which experimental observations can inform prevention or treatment. The contributions collected here approach these questions through cancer signalling, neurodevelopment, endocrine regulation, ageing, comparative toxicology, and respiratory host–microbe interactions.

The collection brings together three original investigations, two narrative reviews, and one systematic review. The overarching theme in these contributions is the relationship between biological mechanism and functional context. Molecular findings acquire meaning through their localisation in cells and tissues, their variation across the life course, and their consequences for the organism. A One Health perspective extends this reasoning to the relationships among human, animal, and environmental health. The contributions address different parts of this broader agenda, together illustrating the opportunities for integration and the need to define the limits of translation.

Porreca et al. reviewed extracellular signal-regulated kinase 1/2 (ERK1/2) signalling in intrahepatic cholangiocarcinoma [1]. They connect pathway dysregulation with tumour growth, invasion, and interactions with the microenvironment, and the authors discusses pharmacological approaches targeting the signalling cascade. This review provides a clear example of how mechanistic research identifies therapeutic opportunities while also revealing obstacles, including molecular heterogeneity and resistance. Evidence from experimental systems and early clinical investigations supports further study but does not establish routine clinical efficacy of ERK1/2 in this disease. The inhibition of upstream pathway components must be distinguished from direct ERK inhibition when interpreting the therapeutic literature. The translational question is therefore how to identify the biological contexts in which pathway targeting produces a durable benefit.

The importance of the normal biological context is illustrated by Demirtaş et al., who investigated artemin protein and *Gfra3* messenger RNA in the prefrontal cortex, striatum, and cerebellum of male rats at three postnatal ages [2]. The cerebellar artemin levels were higher than those in the other regions, whereas significant age-related variation in artemin levels was not detected. The cerebellar *Gfra3* expression level varied with age and was highest at the intermediate time point. These observations provide a regional and developmental reference for subsequent functional studies. The authors also underscore a distinction central to experimental biology: measuring ligand abundance and receptor transcripts does not, by itself, describe signalling activity or establish a causal role in maturation. Cell-specific localisation and functional perturbation would help explain the identified patterns.

Koçak et al. connected endocrine measurements with tissue immunoreactivity and behavioural outcomes in a methimazole-induced hypothyroidism rat model [3]. Spexin administration reduced thyroid-stimulating hormone and increased thyroxine concentrations, while attenuating reductions in irisin and KISS-1 immunoreactivity in the cerebral cortex and testis. The behavioural findings were more circumscribed: changes in thigmotaxis were found, but overall locomotor activity and forced-swim outcomes did not significantly differ among groups. This combination of findings encourages the investigation of spexin-associated neuroendocrine regulation without implying a general behavioural recovery or an established therapeutic alternative. The study also illustrates why biochemical, morphological, and functional endpoints should be interpreted together: their agreement can strengthen a hypothesis, whereas their differences help define its scope.

Moving from development and endocrine regulation to ageing, Kim and Kim systematically reviewed swallowing-related alterations in healthy aged rodents [4]. Their synthesis of 29 studies encompasses muscle contractile properties, histomorphological and biochemical features, cranial sensorimotor systems, and swallowing behaviour. Functional decline is placed within a distributed neuromuscular system rather than attributing it to a single structure. For translational research, the value of these models lies in the possibility of relating tissue-level changes to measurable aspects of swallowing performance. Additionally, the anatomical and biomechanical differences between rodents and humans, together with the variability among experimental approaches, must inform model interpretation. Understanding healthy ageing provides an essential reference against which disease-associated dysfunction and potential preventive or rehabilitative interventions can be evaluated.

Sukaj et al. provide a direct connection between comparative biology and environmental health through their investigation of zinc oxide nanoparticles [5]. They compared nucleated frog erythrocytes with anucleate human erythrocytes, finding different patterns of injury: prominent membrane damage and haemolysis in human cells and DNA damage with relatively preserved membrane integrity in frog cells. Experimental assessments were complemented by molecular docking studies. The study demonstrates the value of examining several biological endpoints across species when characterising a potentially harmful exposure. Nevertheless, species and nuclear status vary together in this comparison, so the findings should not be exclusively attributed to the presence or absence of a nucleus. Likewise, docking offers hypotheses about molecular interactions, whereas organism-level risk assessment requires additional evidence on exposure, distribution, and biological effects.

Finally, Candia et al. considered another interface between biological systems in their review of the respiratory muco-microbiotic (MuMi) layer [6]. The proposed framework brings mucus, resident microorganisms and extracellular vesicles into a shared functional context at the epithelial surface. By connecting transcriptomic and metatranscriptomic activity with inflammatory phenotypes and candidate biomarkers, the authors outline how this interface could inform precision pulmonology. Their contribution is conceptual and integrative: they encourage the investigation of microbial activity and host–microbe communication alongside community composition. Prospective studies and reproducible sampling strategies will be important for determining which MuMi-associated measurements add clinically useful information. This perspective complements the toxicological contribution by locating interactions with the surrounding environment within a biologically active tissue interface.

Read together, these papers suggest that the tissue context should remain central as experimental biology becomes increasingly molecular and computational. A concentration measured in a homogenate, a transcript profile, an immunohistochemical signal, and a behavioural response describe different aspects of a system. Linking them requires attention to sampling, spatial organisation, and the biological question each method can answer. Histology can help connect molecular observations with the cells and compartments in which they arise; functional experiments can then test whether these observations explain changes at the organ or organism level. This relationship is particularly relevant when the aim is to identify a biomarker or therapeutic target.

This collection also encourages a precise use of the term “translational”. Descriptive developmental data, comparative toxicity experiments, and mechanistic reviews can all contribute to translation, but they occupy different positions along the path towards practical application. Their next steps will therefore differ: localisation and perturbation studies for expression patterns, independent validation for candidate biomarkers, exposure-relevant testing for toxicological findings, and appropriately designed clinical investigations for therapeutic hypotheses. Explicitly identifying these steps clarifies the contribution of basic research and helps prevent promising observations from being mistaken for validated interventions.

Similarly, the One Health ambition of this Special Issue is best understood as a direction for integration. Most contributions focus on biomedical mechanisms or animal models, whereas comparative toxicology and the respiratory host–microbe interface provide more explicit connections with environmental questions. Future work can strengthen our understanding of these connections by examining shared exposures, species-specific susceptibility, and the effects of environmental change on biological function. Such studies would benefit from collaboration across experimental biology, clinical medicine, veterinary science, and environmental research, with each discipline contributing methods suited to the question being addressed.

A century after the foundation of the Italian Society of Experimental Biology, the work assembled in this Special Issue illustrates the continuing relevance of an approach that connects observation with experiment and mechanism with function. Its lasting value will lie in the questions this Special Issue helps refine and the investigations it enables. By preserving biological context and clearly distinguishing evidence from expectation, experimental biology can continue to support a more integrated understanding of health and a more rigorous progression from basic discovery to translational research.
